# Genetic diversity and molecular characterization of enteroviruses from sewage-polluted urban and rural rivers in the Philippines

**DOI:** 10.1007/s11262-012-0776-z

**Published:** 2012-06-29

**Authors:** Lea Necitas G. Apostol, Tomifumi Imagawa, Akira Suzuki, Yoshifumi Masago, Socorro Lupisan, Remigio Olveda, Mariko Saito, Tatsuo Omura, Hitoshi Oshitani

**Affiliations:** 1 Department of Virology, Tohoku University Graduate School of Medicine, 2-1 Seiryo-machi, Aoba-ku, Sendai 980-8575 Japan; 2 Research Institute for Tropical Medicine, Filinvest Corporate City Compound Alabang, 1781 Muntinlupa, Philippines; 3 Tohoku-RITM Collaborating Center for Emerging and Reemerging Infectious Diseases, Muntinlupa, Philippines; 4 Department of Civil and Environmental Engineering, Tohoku University Graduate School of Engineering, 6-6-06 Aoba Aramaki, Aoba-ku, Sendai, 980-8579 Japan

**Keywords:** Enterovirus, Environmental surveillance, Molecular epidemiology, Porcine enterovirus, Genetic diversity

## Abstract

Despite the vast distribution and expansive diversity of enteroviruses reported globally, indicators defining a complete view of the epidemiology of enteroviruses in tropical countries such as the Philippines are yet to be established. Detection of enteroviruses in the environment has been one of the markers of circulating viruses in a community. This study aimed to bridge the gap in the epidemiology of enteroviruses in the Philippines by providing an overview of the occurrence of enteroviruses in both urban and rural rivers. Molecular detection directed at the VP1 region of the enterovirus genome was performed on 44 grab river water samples collected from April to December 2009. The majority of the enterovirus serotypes detected were clustered with human enterovirus C species (HEV-C; 21/42), followed by HEV-B (12/42) and HEV-A (9/42). Porcine enterovirus 9 was also found in 12 out of 44 water samples. Phylogenetic analysis indicated that the viruses detected were closely related, if not all forming a monophyletic clade, with those enteroviruses detected previously from acute flaccid paralysis cases in the country. The clustering of environmental and human enterovirus strains implies that the circulation of these strains were associated with river contamination. This study gives further evidence of the environmental persistence of enteroviruses once they are shed in feces and likewise, provides additional data which may help in understanding the epidemiology of enteroviruses in humans, highlighting the need for more studies on the potential public health risks linked with enteroviruses found in the environment and their eventual clinical consequences in the country.

**Electronic supplementary material:**

The online version of this article (doi:10.1007/s11262-012-0776-z) contains supplementary material, which is available to authorized users.

## Introduction

Enteroviruses (EVs) of the family *Picornaviridae* have been implicated as viral agents associated with numerous clinical presentations in humans, ranging from asymptomatic to life-threatening diseases [1–3]. Significant public health impacts have been attributed to some EVs for large outbreaks of hand-foot-mouth disease, debilitating poliomyelitis, aseptic meningitis, encephalitis, and keratoconjunctivitis [4–7]. EVs are also implicated as a potential cause of chronic diseases such as insulin-dependent diabetes mellitus and dilated cardiomyopathy [1, 8]. Nearly all EVs replicate in the gastrointestinal tract of humans and large amounts, ranging from 2.0 to 6.5 log10 TCD50 per gram of stool or 10–300 million TCD50 of virus, of enteroviral particles are shed daily in the feces of an infected individual. Transmission takes place mainly by fecal–oral route [9]. Since EVs contain no lipid envelope, these viruses are resistant to ether, chloroform and other lipid solvents and can persist under harsh environmental conditions such as extreme pH and temperature rendering them infective for long periods [10–12]. This biological attribute of the persistence of EVs in both terrestrial and aquatic environments supports sustained circulation. Owing to its uninterrupted spread, various EV serotypes may have undergone genomic modifications by means of mutations and recombinations, giving rise to the emergence of novel EVs. The conservation of this virus in nature permits constant widespread circulation warranting laboratory monitoring or surveillance of EVs in water environments.

Worldwide, the epidemiology of EVs is examined by specific studies of human populations, which describes both temporal and disease patterns [13–17]. Besides isolation of infective virions, assessment on the probable significance of viral genes that persist in water has also been used to establish a correlation with the disease dynamics in humans. This approach has been applied to poliovirus (PV) studies and outlines our present understanding of the eclectic epidemiological links that stretch beyond human reservoirs [18–21]. In developing countries, data on prevalent and circulating pathogenic organisms such as EVs, and other gastrointestinal viruses is limited due to lack of an adequate surveillance system. Detection of these viruses in river waters and sewage can be an alternative method to monitor circulating viruses in humans [22–27]. Most viruses found in sewage and river waters are common enteric pathogens including EVs with concentrations reaching as high as 2.3 × 10^6^ particles per milliliter and 1,390 PCR-detectable units/l, respectively [28, 29]. Several reports have described water contaminated with human EVs, especially in sites close to urban areas and related to outbreaks of human infections [30–32].

Understanding the distribution and persistence of EVs in sewage-contaminated river waters from different geographical areas may provide relevant information on the epidemiology of enteroviral infections circulating in the community, especially in resource-limited settings. Guidelines on environmental monitoring of EVs in the context of the poliomyelitis eradication initiative have been drafted and states that environmental monitoring can be a complementary tool to existing acute flaccid paralysis (AFP) surveillance systems especially in urban settings [33]. Some countries have already adapted environmental PV surveillance. Evidently, these systematic surveillances have offered supplementary information on the circulation of PVs and the emergence of vaccine-derived PVs [18, 34]. In countries declared polio-free, clinical, and epidemiological investigations as well as environmental surveys outlining the presence of EVs in sewage-polluted rivers have been documented [35, 36]. However, the impact of aquatic transmission in flow waters is not well understood especially for EVs except for PVs. In a study of children, it was noted that higher relative risk was found among those who happened to be beach swimmers suggesting that accidental drinking of contaminated water may be a means of spreading EV infection [37]. In developing countries, children’s behaviors such as swimming or playing in rivers are a common scenario wherein, inadvertently, children may swallow river water that contains the infective viral dose range of 30–1,000 EV particles [38]. Given that a low amount of virions can infect a susceptible individual; this practice can be regarded as a potential exposure to contract EV infection especially in low socioeconomic areas such as the Philippines. In addition, not much is known about whether EVs circulate in the environment and their eventual associations with neurological, diarrheal or chronic illnesses in developing countries.

In the Philippines, the epidemiology of EVs remains incomplete because EV isolation and detection have been performed so far only on isolates from AFP samples and to our knowledge, there are no reports of EV detection in environmental samples [39]. With the assertion that river water samples may reflect the prevalent EVs circulating in the Philippines, in this study, we examined environmental samples to describe the occurrence and diversity of EV types in major sewage-polluted urban and rural rivers using molecular detection and sequence analysis of viral genes.

## Materials and methods

### Sample collection

**Fig. 1:**
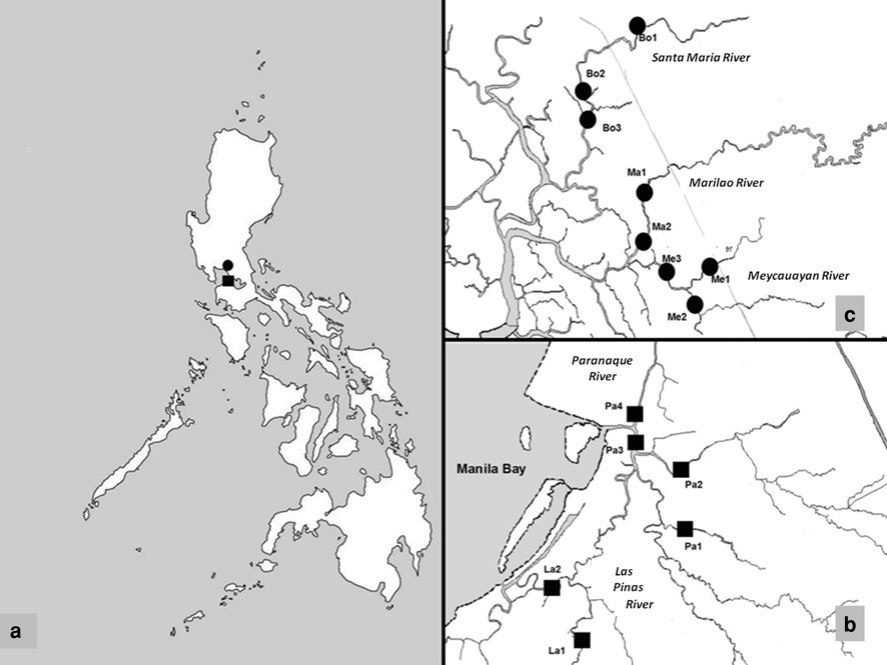
Sampling sites. **a** Philippine map. **b** Metro Manila (*filled square* urban, La—Las Pinas, Pa—Paranaque). **c** Bulacan (*filled circle* rural, Bo—Bocaue, Ma—Marilao, Me—Meycauayan)

A total of 44 grab water samples from sewage were collected from six and eight sampling sites in major urban cities (Metro Manila: Las Pinas, Paranaque) and neighboring rural towns (Bulacan: Bocaue, Marilao, and Meycauayan), respectively (Fig. 1). Samples (2000 ml) were collected at different sites of urban and rural river waters that receive sewage from a population of 1,084,990 and 462,838 people, respectively (http://www.census.gov.ph/data/census2007/h030000.pdf). Metro Manila has a population density of 18,246 people per square kilometer while Bulacan has 1,077 people per square kilometer. Water samples were drawn at all locations for at least three times from April to December 2009. The water samples were kept at 4 °C during transport and then taken to the Research Institute for Tropical Medicine for processing.

### Concentration and purification of river water samples

Water samples were concentrated by precipitation with polyethylene glycol 6000 (PEG 6000) and sodium chloride (Wako Industries, Osaka, Japan) to a final concentration of 8.0 and 2.1 % (wt./vol.), respectively. The resulting suspension was stored at 4 °C overnight and centrifuged at 3,220×*g* for 1 h at 4 °C. Subsequently, the PEG-containing supernatant was decanted and the pellet was suspended in 3 ml phosphate-buffered saline (PBS) at pH 7.0 and filtered with 0.45 μm pore filter (Advantec, CA, USA). The sample concentrates were then stored at −80 °C until RNA extraction.

### Viral amplification, cloning, and sequencing

Total viral RNA was extracted from 200 μl of sample concentrate using Purelink Viral DNA/RNA kit, according to manufacturer’s instructions and reverse-transcribed with M-MLV using random primers, with all reagents purchased from Invitrogen (CA, USA) unless specified.

For porcine enterovirus (PEV) detection, we utilized a reverse-transcription polymerase chain reaction (RT-PCR) that had been optimized for the specific detection of PEV as described previously [40]. The protocol targets the highly conserved 5′-untranslated region (UTR) of PEVs. For EVs, we employed an RT-PCR on fragments encompassing the highly conserved part of the viral polyprotein 1 (VP1) region (Table 1), as described previously [40–42]. The amplicons were analyzed by electrophoresis using 1.5 % agarose gels and visualized under UV illumination following ethidium bromide staining.

**Table 1:** Primers used in this study

Primer ID	Sequence (5′–3′)	Product length (bp)	Primer position
*Enteroviruses*			
292	MIGCIGYIGARACNGG	340	nt 2612–2627
222	CICCIGGIGGIAYRWACAT		nt 2969–2951
*Porcine enteroviruses*			
PEV-1F	GTACCTTTGTACGCCTGTTTTA	491	nt 66–87
PEV-1R	ACCCAAAGTAGTCGGTTCCGC		nt 556–536
PEV-NF	CAAGCACTTCTGTTTCCCCGG	313	nt 167–187
PEV-NR	GTTAGGATTAGCCGCATTCA		nt 479–460

Direct sequencing was performed for samples with a single and specific band from both directions with the ABI Big Dye Terminal Cycle Sequencing kit version 3.1 according to manufacturer’s instructions and ABI Prism 3730 genetic analyzer (Applied Biosystems, CA, USA). However, an initial attempt was unsuccessful due to the existence of genes from multiple EVs in the sample. Therefore, molecular cloning was performed on all amplicons produced by primers 292 and 222 using the pGEM-T vector system (Promega Corporation, USA) and the construct was transformed into JM109 *Escherichia coli* competent cells (Promega Corporation, USA). All white colonies/populations were selected and primed with the same primers used in RT-PCR for VP1 region. Next the PCR products were purified using the Wizard SV Gel and PCR Clean-up system (Promega Corporation, USA) prior to sequencing.

The nucleotide sequences were assembled, aligned and analyzed in MEGA version 5.0 and compared with reference strains deposited in GenBank database and from a previous study on EVs in the Philippines [39]. Multiple alignments of the VP1 region for HEV and of the 5′-UTR of PEV were done by Clustal W and phylogenetic analyses for each serotype determined were performed by neighbor-joining method. Phylogenetic distances were calculated using Kimura-2-parameter method and the reliability of the trees was estimated by means of a bootstrap analysis of 1,000 pseudoreplicates.

### Nucleotide sequence accession numbers

The nucleotide sequences determined in this study were deposited in GenBank database under the following accession numbers, JQ744283-JQ744336.

## Results

### Distribution and diversity of EVs in river waters

Molecular identification directed at the VP1 region for EVs allowed the detection of 63.6 % in grab river water samples (28/44), 60.0 % (12/20) in urban Metro Manila and 66.7 % (16/24) in the rural Bulacan sites (Table 2). Identification of PEV using primers that target the 5′-UTR yielded the expected amplicon size for 12 out of 44 samples (five from Bocaue, four from Marilao, two from Meycauayan and one from Paranaque) and direct sequencing revealed that the 5′-UTR sequences indicated PEV9 (Table 3). For the VP1 region, although 28/44 samples showed the expected amplicon size, direct sequencing of the PCR products to obtain nucleotide sequence reads could not be determined, suggesting that multiple EV genes were present in the sample. All 28 positive amplicons in the VP1 region were then subjected to molecular cloning and resulted in the identification of various EVs present in a single sample.

**Table 2:** Detection of enteroviruses from river waters, Philippines, 2009

	Site	%	Simple ID	Date collected	Clones	HEV-A(9)				HEV-B(12)						HEV-C(21)						
		Positive				EV 71	CV A3	CV A8	CV A16	El 1	E21	EV 80	CV B6	CV B5	CV B2	CV A17	CV A20	CV A21	CV A24	EV 96	EV 99	Total
Metro Manila (Urban)	LP	5/6 (83.3 %)	2	Apr	14												1					1
			19	Aug	7						1							2				3
			27	Aug	9				1	3												4
			33	Dec	5														2			2
			34	Dec	7							1										1
	Pa	7/14 (50.0 %)	3	Apr	6	1																1
			20	Aug	10														1	1		2
			21	Aug	6		2															2
			22	Aug	5														1			1
			23	Aug	7												1					1
			35	Dec	5						1										1	2
			36	Dec	13		1	1						1	1	2						6
		Subtotal	12/20 (60.0 %)		104	1	3	1	1	3	2	1	0	1	1	2	2	2	4	1	1	26
Bulacan (Rural)	Bo	5/9 55.6 %	6, 7, 8	Apr	Not done (ND)																	
			29	Aug																		
			44	Dec																		
	Ma	5/6 (83.3 %)	12	Apr	4							1										1
			30	Aug	6			1											2			3
			37	Dec	5												1					1
			42	Dec	8													1				1
			43	Dec	10	1																1
	Me	6/9 (66.7 %)	24	Aug	7								2			1						3
			28	Aug	ND																	
			31	Aug	4		1															1
			38	Dec	7												1		2			3
			39	Dec	4												1					1
			40	Dec	4						1											1
		Subtotal	16/24 (66.7 %)		59	1	1	1	0	0	1	1	2	0	0	l	3	1	4	0	0	16
All sites		Total	28/44 (63.6 %)		163	2	4	2	1	3	3	2	2	1	1	3	5	3	8	1	1	42

*LP* Las Pinas,* Bo* Bocaue,* Ma* Marilao,* Me* Meycauayan,* EV* enterovirus,* E* echovirus

**Table 3:** Detection of porcine enterovirus (PEV) from river waters, Philippines, 2009

	Site	Positivity rate	Sample ID	Date collection	PEV9 (5′UTR)
Metro Manila (urban)	Las Pinas	0/6			
	Paranaque	1/14 (7.1 %)	22	Aug	1
Bulacan (rural)	Bocaue	5/9	6,7,8	Apr	
		(55.6 %)	29	Aug	5
			44	Dec	
	Marilao	4/6	12	Apr	
		(66.7 %)	37,42,43	Dec	4
	Meycauayan	2/9	28	Aug	2
		22.2 %	40	Dec	
All sites	Total	12/44(27.3 %)			12

The VP1 PCR products of the 28 samples were ligated and inserted into competent cells and a total of 163 clones (104 = urban; 59 = rural) were harvested; 42 clones were positive for the expected amplicons size by PCR (Table 2).

### Phylogenetic analysis of PEV 9 at the 5′-UTR and human EV strains at the VP1region

**Fig. 2:**
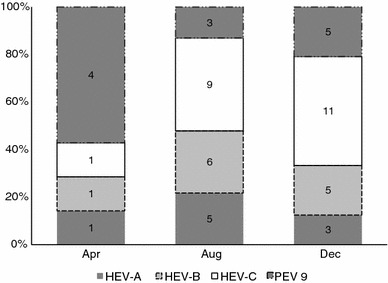
Proportion of enterovirus genotypes from river water detected by month. *HEV-A* human enterovirus A, *HEV-B* human enterovirus B, *HEV-C* human enterovirus C, *PEV* porcine enterovirus

Since the standard for EV identification utilizes sequences of the VP1 region, the present study was able to document the existence of the following serotypes [EV71, Echovirus (E)11, Coxsackievirus B6 (CVB6), CVB5, CVB2, EV80, E21, CVA3, CVA6, CVA16, CVA17, CVA20, CVA21, CVA24, EV96, and EV99]. Identification of EV serotypes in this region was made by a priori molecular cutoff of 15 % nucleotide divergence from BLAST analysis. With this cutoff limit, 42 clones were identified and classified into 16 serotypes of HEV. The most frequently detected serotype belonging to HEV-C species was CVA24, which accounted for 40.0 % (8/20), followed by 25.0 % of CVA20 (5/20) (Table 2; Fig. 2).

**Fig. 3:**
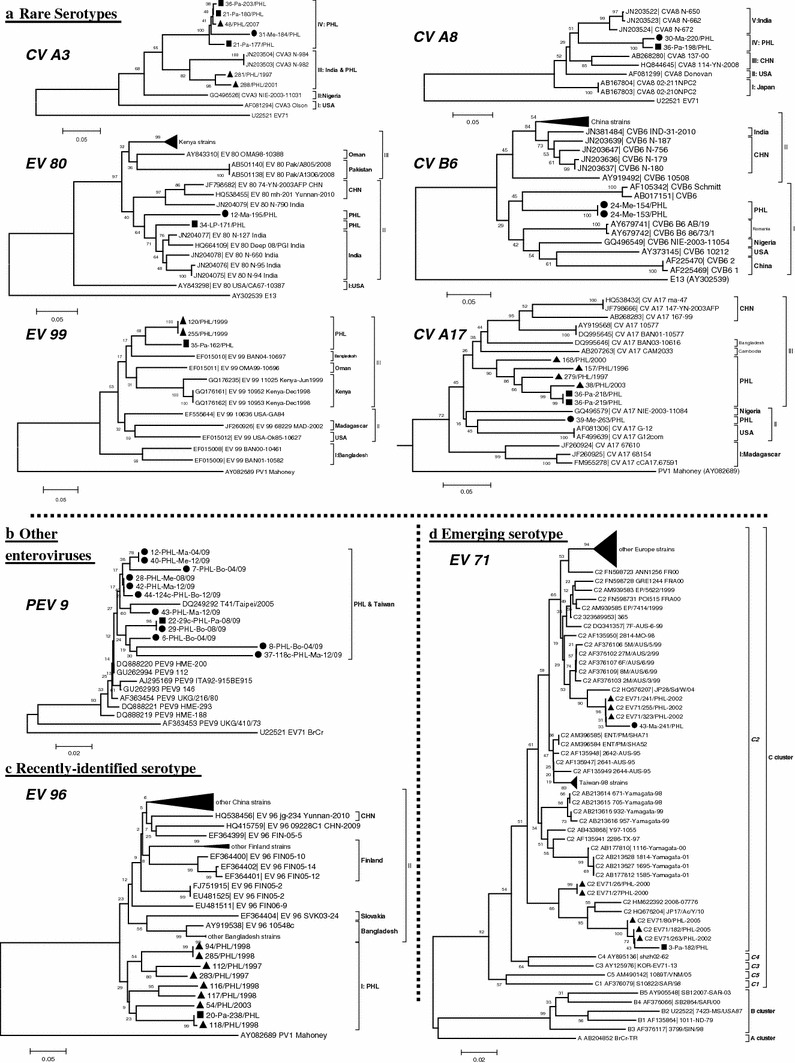
Phylogenetic relationships of selected enterovirus serotypes detected in this study, Philippines, 2009. **a** Rare serotypes. **b** Other enteroviruses. **c** Recently identified serotype. **d** Emerging serotype. Phylogenetic trees were constructed for each serotype by the neighbor-joining method implemented in MEGA 5.0. *Filled triangle* EV strains detected from stool samples in acute flaccid paralysis surveillance in the Philippines. *Filled square* strains detected from this study (urban river samples). *Filled circle* strains detected from this study (rural river samples). *EV* enteroviruses, *CV* coxsackieviruses, *PEV* porcine enteroviruses

The study revealed that several EV serotypes have been identified as belonging to HEV-A (four serotypes), HEV-B (six serotypes), and HEV-C (six serotypes). The majority of the clones that were positive for the expected PCR product size belonged to the HEV-C group (21/42, 50.0 %) followed by HEV-B (12/42, 28.6 %) and HEV-A (9/42, 21.4 %). Although the detection frequency of EV in rural sites was slightly higher than in urban sites (66.7 and 60.0 %, respectively), more EV serotypes were detected in urban river water samples (15 HEVs and 1 PEV) than in rural river samples (10 HEVs and 1 PEV) as shown in Tables 2 and 3. The occurrence of all genotypes of EVs as well as PEV9 was observed in three collection periods suggesting that EVs occured all year round (Fig. 2). Unique serotypes found only in urban rivers were CVA16, CVB5, CVB2, E11, EV96, and EV99; the rest of the detected EVs appeared to be circulating in both urban and rural flow waters except CVB6 which was detected only in rural flow waters (Fig. 3; Supplementary Fig. 1).

Phylogenic analysis of partial 5′-UTR of PEV 9 in the Philippines is shown in Fig. 3b. The tree showed that PEVs detected from rivers in the Philippines (PHL strains) clustered within a single clade with one strain from Taiwan (TAI strain: DQ249292) interspersed with the Philippine PEV9 strains. Due to the small number of sequences of PEV9 deposited in GenBank, inference on their epidemiological link cannot be made.

We recently reported data on EVs from AFP surveillance in the Philippines [39]. Comparison with some of the EV sequences sampled from the aforementioned surveillance in the Philippines revealed that EVs in the rivers were closely related to some strains detected from AFP surveillance (Fig. 3; Supplementary Fig. 1). The trees showed evidence that most of the EV strains found in this study clustered within the known types of circulating EVs previously deposited in GenBank. Rare serotypes with fewer than 20 sequences deposited in GenBank were also documented (Fig. 3a: CVA3, CVA8, EV80, CVB6, CVA17, and EV99). With drawbacks concerning the inference of these rare isolates, however, CVA3 and CVA8 showed at least high homology, both in the phylogenetic tree and by BLAST analyses, to the Indian strains. The closest matches of the CVA3 Philippine strains were the contemporary Indian isolates, JN203503 and JN203504. These Indian isolates were derived from human stool samples from AFP surveillance. Currently, there are only nine available sequences deposited in GenBank for CVA3, five of which are not suitable for alignment in our study because of either different genomic regions or since the length of the VP1 region was either in the upstream or downstream regions of our target primer sequences. Similarly, strains of CVA8 clustered with the strains detected from India and China. Noteworthy, in this study, the Philippine CVA17 strains discovered from both humans and the environment formed a monoclade, designated as clade III, except for one sample (39-Me-263-PHL) which was grouped with USA and Nigerian CVB6 clade (clade II). The isolates of EV80 belonged to the same genetic lineage with clades identified in countries such as India and China that were isolated and detected in 2011 and 2010, respectively. In the same way, the Philippine CVB6 lineage indicated that it was most closely linked to the cluster of CVB6 strains in Romania, Nigeria, USA, and China (clade I). On the other hand, EV99 demonstrated a close relationship with strains from Bangladesh, Oman and Kenya.

In this study, detection of prevalent serotypes such as CVB2, CVB5, E11, CVA24, and CVA21, appeared to have a large-scale circulation worldwide as they are closely related to isolates identified from different countries (Supplementary Fig. 1). Remarkably, although the detection of CVA20 in the Philippines showed two clusters (CVA20, clades II, and V; Supplementary Fig. 1), two river samples and four strains from humans previously reported in Philippines formed a single clade, clade II. Similarly, E21 belonged to the clade V, which is composed of China, India and Bangladesh strains (Supplementary Fig. 1). In comparison with the available sequences of EV96 in GenBank, the EV96 strain found in this study formed a distinct monoclade, clade I, with previously reported strains of EV96 from AFP cases in the country (Fig. 3c).

Notably, some important emerging EV pathogens that cause hand-foot-mouth disease such as EV71 and CVA16, were also detected in this study and their sequences were compared with the available sequences deposited in GenBank. Enterovirus 71 was grouped with the existing EV71 subgenogroup C2 in the country. Strain 3-Pa-182/PHL was collected from an urban river clustered with human EV71 strains also isolated from the same city (C2 EV71/263/PHL-2002, C2 EV71/182/PHL-2005, C2 EV71/80/PHL-2005), whereas strain 43-Ma-241/PHL grouped with EV71 Philippine strains was isolated from Regions IV (C2 EV71/323/PHL-2002) and VIII (C2 EV71/241/PHL-2002 and C2 EV71/255/PHL-2002) (Fig. 3d). Comparison of the CVA16 Philippine strain found in this study identified it as belonging to the cluster of CVA16 strains found in China and Korea (Supplementary Fig. 1, CVA16).

## Discussion

We detected a multitude of EV serotypes from environmental samples collected from urban and rural flow waters in the Philippines. The present study revealed a relatively higher detection frequency (66.7 %) in rural river water samples than in urban samples. Although urban samples had a lower positivity rate for EVs, a vast genetic diversity of EV strains was detected. The relative abundance and diversity of EVs observed in urban rivers might be a reflection of the current situation in Metro Manila where population density is higher than in rural areas.

Monitoring of pathogenic microorganisms including viruses in natural environments such as flow waters in correlation with clinical cases in a community is valuable for possible prediction of outbreaks [43]. But due to lack of evidence or studies on the risk factors of EV infection in the Philippines, no substantial basis is tangible to develop effective prevention and control strategies against enteroviral infections. However, in this study, we found that prevalent EVs detected from the environment mirror the currently circulating EVs in the community/population. This data, therefore, can be used as one of the indicators for understanding EV circulation in the community.

While a complete VP1 gene sequence is the best to identify EV serotypes, the study relied on using highly conserved 292/222 primers that target a part of the VP1 region, the gene that encodes serotype-specific neutralization epitopes and that is used for EV classification [44]. In this study, although partial VP1 sequences were amplified, the study showed the variability of HEV strains present in river water samples and all the detected EVs in this study have also been found in human samples from AFP cases. Furthermore, the study found a detection frequency pattern, HEV-C > HEV-B > HEV-A > PEV, which contrasted with the recent study sampled from AFP surveillance where abundance of the pattern HEV-B > HEV-C > HEV-A was documented [39]. Whether the pattern documented in this study covering the environment or the pattern shown by the recent study of human AFP cases is the true picture cannot be discerned at this point. Further studies on a much larger scale for environmental surveillance as well as a broader spectrum of diseases caused by EVs in the human population should be made available for comparison. Although the study was based on molecular detection and not viral isolation, the finding that HEV-C viral RNA is the predominant genotype left a question of whether the survival of HEV-C in rivers is indicative of its better adaptation and resistance to the environment than HEV-B. Then again, this view should be regarded only as indicative since no reports have suggested or confirmed the possibility of this claim. Even so, the results provided in this analysis support a recent study in Singapore where genotype C is also the dominant type in environmental samples [45]. Although a larger sample size is required to draw a definitive conclusion on the presence of EV infection in the study area, the high proportion of HEV-C implies a wide spread of EVs among urban and rural dwellers. In addition, the observation that all genotypes can be found in both urban and rural rivers indicates the expansive circulation of the EVs in general.

PEVs have been isolated from swine having asymptomatic infections in many regions including Italy [46]. In this study, the detection of PEVs in all Bulacan towns (rural) and in Las Pinas (urban) river waters demonstrates the possibility that viruses come from swine farms that abound in both areas. Paranaque, though located in an urban district, is one of the southernmost areas in Manila and is close to the province of Calamba where a lot of pigs are farmed. It has been documented that swine serve as animal hosts for EVs to perpetuate in humans.

While samples showed the expected amplicon size in the PCR, some samples could not be identified in sequencing simply because of a mixed viral population of the same family *Picornaviridae* present in the sample. The cloning of PCR products showed that some of the samples harbored diverse EV types with related but non-homologous sequences. The finding is significant because it exposes the occurrence of numerous viral genomes and the genetic disparity in a single pool of samples.

Similar to other RNA viruses, genomic variability among EVs is very high and therefore, permits the generation of new variants within a community. The vast diversity of EV and the eclectic reservoirs for EVs, especially for HEV-C, may permit genomic exchanges such as recombination. More studies on the HEV-C strains may offer additional information about recombination events among human EV strains. The erratic and rapid nature of EV evolution along with epidemiological shifting of infections is a matter of consideration. Another risk to achieving global PV eradication is the emergence of circulating vaccine-derived PVs (cVDPVs), PVs in nature that have recombined with HEV-C species. In the Philippines, type 1 cVDPV was involved in three cases in 2001. EV surveillance conducted in human populations and the environment, is also an important tool for monitoring cVDPVs. This study confirms that the relatively high abundance of HEV-C genotypes in the environment provided a significant view of a huge pool of reservoirs for EV recombination to occur. Combining the viral isolates of HEV-C detected in human samples from AFP cases reported recently with the high prevalence of HEV-C species detected in this study will be helpful to identify the donor strain of type 1 cVDPV in the Philippines at the non-structural region [39, 47].

Even though CVA3 and CVA8, among others, were categorized as rare serotypes due to small number of sequences available for comparison, their relatively high detection frequency in the Philippines is remarkable. The phylogenetic analyses performed for these rare serotypes were based on the alignment of less than 20 sequences derived from GenBank and so are not sufficient to infer the relative distribution of these isolates in the region (Fig. 3a). Conversely, the clustering of most of the Philippine EV strains with Asian strains detected in this study may suggest that their circulation is widespread throughout the region.

Aside from the fact that there is growing detection of new EVs worldwide, and since their potential health impact has not been documented at large, the emergence of new EVs such as EV96 or EV99 poses potential risks to the community as these EVs may be better equipped to infect susceptible individuals. This study further supports the idea that EVs can persist in harsh environmental conditions. It is still unknown whether these viral RNAs correspond to infectious viruses because only the viral RNA is detected and not isolated from the samples. Correlation studies together with human population studies with a much wider coverage of diseases are warranted in order to elucidate the significance of these findings. Moreover, whereas it may be true that some EVs can be found only in urban rivers, the study is limited to further interpretation since it covered only a year-long period.

The feasibility of conducting environmental surveillance as a tool to clarify the epidemiology and impact of EVs circulating in a community is based on the account that excreted enteroviral particles remain stable in the environment and that these viruses can be detected even at long sampling intervals. The use of environmental samples to verify the circulating viruses in the community, as in this study, is an effective means of providing auxiliary information adjunct with existing epidemiological studies done on human population.

Detecting EVs in waters has been replaced by broader gastrointestinal virus screening including that of norovirus and rotavirus, but in the light of PV eradication, continuous monitoring of non-polio EVs is still significant. Despite the advantages of surveillance in humans such as AFP, conducting surveillance is relatively costly and the coverage, though national in structure, is still flawed since only those who consult hospitals are likely to be enrolled in the surveillance. Thus, data obtained are underestimates. In these circumstances, environmental surveillance is more sensitive and faster in depicting the progress of the disease in a given community. With these downsides, inevitable for most of the national AFP surveillances in geographical locations that are resource-limited like the Philippines, environmental monitoring which is economical and not labor-intensive, can be used as a surrogate marker to detect the presence of enteroviral infections. Moreover, with the average non-polio enterovirus (NPEV) rate of 7.8 % in the Philippines and continuous reporting of outbreaks caused by EVs from neighboring countries, low-cost environmental monitoring seems to be a practical approach to rapidly assess EV disease impact.

In conclusion, this work validates the wide circulation of EVs in the Philippines with a predominance of HEV-C strains in both urban and rural river waters. Moreover, this study, based on a year-long monitoring of river water from 14 sites in major urban and rural areas, revealed that NPEVs are not only common but also very widespread and of considerable genetic diversity. Although the study reported the abundance of enteroviral RNAs, it was the first to provide evidence of the relative frequency and genetic diversity of EVs in flow waters in the Philippines. However, our results highlight the need for further studies to understand the role of these viruses in multiple enteroviral illnesses.

The circulation pattern of EVs differs largely by geography, climate, and season [13, 15, 35]. The information on the epidemiology of EVs in the Philippines is still incomplete since only data on human population has been recently documented. The present study can gradually fill in the gaps by documenting the occurrence of EVs in the tropics particularly in the Philippines.

## Electronic supplementary material

Below is the link to the electronic supplementary material.

Supplementary material 1 (PPTX 192 kb)

Supplementary Fig. 1 Phylogenetic trees of other enteroviruses detected in this study. Phylogenetic trees were constructed for each serotype by the neighbor-joining method implemented in MEGA 5.0. ▲ EV strains detected from stool samples in acute flaccid paralysis surveillance in the Philippines; ■strains detected from this study (urban river samples); ●strains detected from this study (rural river samples). EV, enteroviruses; CV, coxsackieviruses
