# Supplementary figures and images for: Genetic diversity and molecular characterization of enteroviruses from sewage-polluted urban and rural rivers in the Philippines
Source: Virus Genes. 2012 Jun 29;45(2):207–17. doi: 10.1007/s11262-012-0776-z (PMC3448906; doi:10.1007/s11262-012-0776-z)

## Slide 1
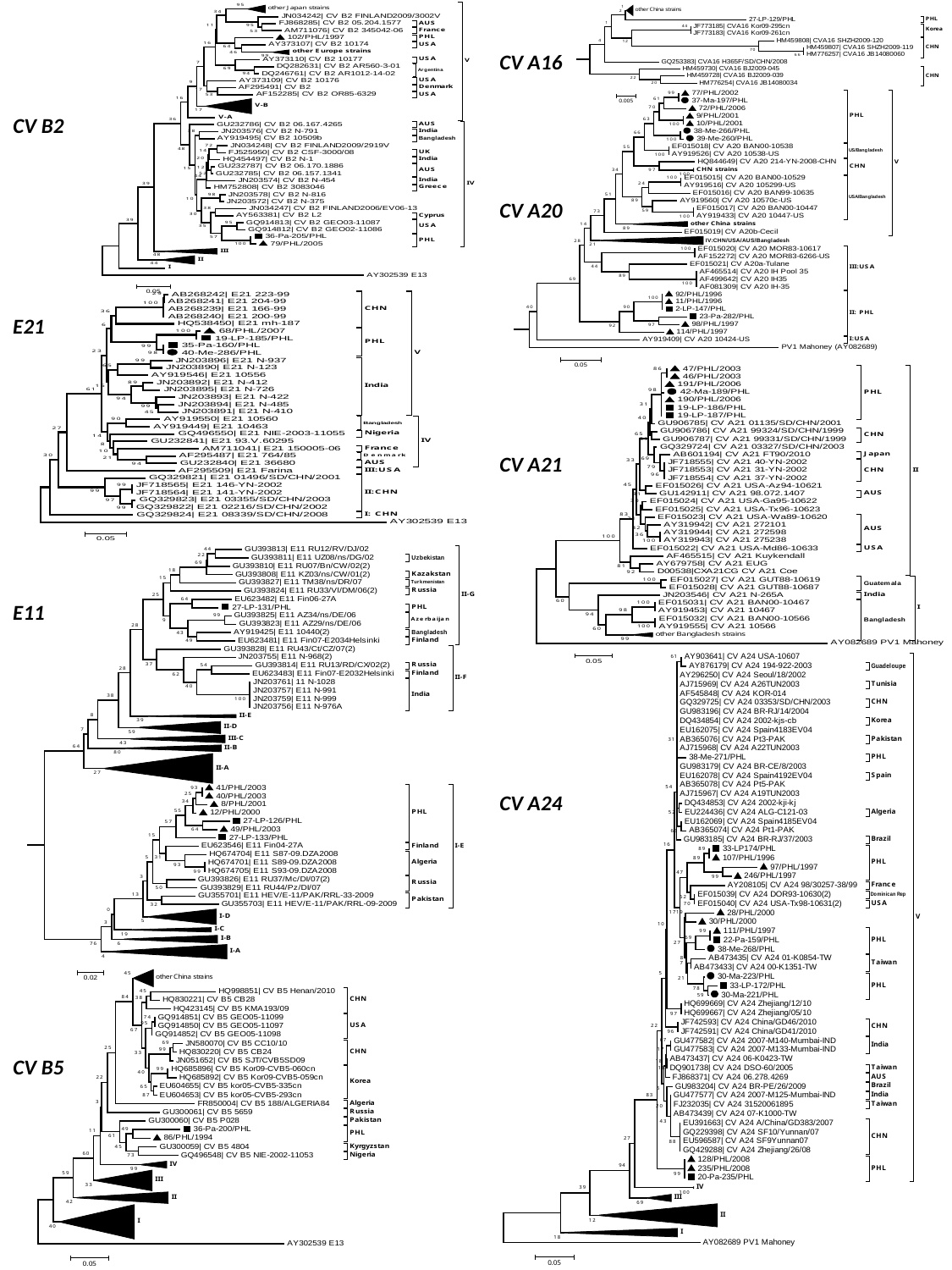

CV A16
CV B2
CV A20
E21
CV A21
E11
CV A24
CV B5

Supplement: Supplementary file 1 — Supplementary material 1 (PPTX 192 kb) [file 11262_2012_776_MOESM1_ESM.pptx]
